# Relationship between coping styles and lipid profile among public university staff

**DOI:** 10.1186/s12944-017-0438-1

**Published:** 2017-02-28

**Authors:** Suthahar Ariaratnam, Ambigga Devi Krishnapillai, Aqil Mohammad Daher, Mohd Ariff Fadzil, Salmi Razali, Siti Aminah Omar, Ng Kien Keat, Nafiza Mat Nasir, Maizatullifah Miskan, Mazapuspavina Md Yasin, Jo Anne Saw, Damayanthi Durairajanayagam, Gurpreet Kaur, Osman Che Bakar, Nurul Azreen Hashim

**Affiliations:** 1 Discipline of Psychological and Behavioural Medicine, Faculty of Medicine, Universiti Teknologi MARA, Kampus Selayang, Jalan Prima Selayang 7, 68100 Batu Caves, Selangor Malaysia; 2 MusTReWell, Brain and Neuroscience Communities of Research, Universiti Teknologi MARA, 40450 Shah Alam, Selangor Malaysia; 3 Department of Primary Care Medicine, Faculty of Medicine and Defence Health, National Defence University of Malaysia, Sg Besi, 57000 Kuala Lumpur, Malaysia; 4 Department of Public Health, Faculty of Medicine and Defence Health, National Defence University of Malaysia, Sg Besi, 57000 Kuala Lumpur, Malaysia; 5 Discipline of Public Health, Faculty of Medicine, Universiti Teknologi MARA, Kampus Sungai Buloh, Jalan Hospital, 47000 Sungai Buloh, Selangor Malaysia; 6 Discipline of Psychological and Behavioural Medicine, Faculty of Medicine, Universiti Teknologi MARA, Kampus Sungai Buloh, Jalan Hospital, 47000 Sungai Buloh, Selangor Malaysia; 7 Discipline of Primary Care Medicine, Faculty of Medicine, Universiti Teknologi MARA, Kampus Selayang, Jalan Prima Selayang 7, 68100 Batu Caves, Selangor Malaysia; 8 Discipline of Physiology, Faculty of Medicine, Universiti Teknologi MARA, Kampus Sungai Buloh, Jalan Hospital, 47000 Sungai Buloh, Selangor Malaysia; 9 Secretariat of National Institutes of Health, Ministry of Health Malaysia, c/o Institut Pengurusan Kesihatan, Jalan Rumah Sakit Bangsar, 59000 Kuala Lumpur, Malaysia

**Keywords:** Coping styles, Lipid profile, Coping Inventory for Stressful Situations, Cardiovascular disease, Biochemical marker

## Abstract

**Background:**

The scarcity of data about coping styles with a biochemical marker namely lipid profile, potentially associated with cardiovascular risk factors is most striking among professionals working in public university. Hence, this research aimed to investigate the relationship between coping styles and lipid profile comprising total cholesterol (TC), triglyceride (TG), HDL-cholesterol (high density lipoprotein-cholesterol) and LDL-cholesterol (Low density lipoprotein-cholesterol) among this group of professionals.

**Methods:**

A cross sectional survey was conducted among staff from a tertiary education centre. Subjects were contacted to ascertain their medical history. A total of 320 subjects were interviewed and 195 subjects were eligible and subsequently recruited on a suitable date for taking blood and administration of the questionnaires. The subjects completed questionnaires pertaining to demographic details and coping styles. Pearson’s correlation coefficient was used to measure the strength of association between lipid profile and coping styles.

**Results:**

Majority of the subjects were non-academic staff (60.0%), female (67.2%), Malay (91.8%), married (52.3%) and educated until Diploma level (34.9%). Academic staff scored significantly higher mean scores in task-oriented coping styles (Mean = 64.12). Non-academic staff scored significantly higher mean scores in emotion (Mean = 48.05) and avoidance-oriented coping styles (Mean = 57.61). Malay subjects had significantly higher mean scores in emotion (Mean = 47.14) and avoidance-oriented coping styles (Mean = 55.23). Non-malay subjects (Mean = 66.00) attained significantly higher mean scores in task-oriented coping styles. Single/divorced/widowed individuals scored significantly higher mean scores in emotion (Mean = 48.13) and avoidance-oriented coping styles (Mean = 56.86). There was a significant negative correlation between TC (*r* = −0.162) and LDL (*r* = −0.168) with avoidance-oriented coping styles (*p* = 0.023, *p* = 0.019 respectively).

**Conclusion:**

Avoidance-oriented coping style was more likely to engender favourable lipid profile. Hence, assessment of coping styles would certainly assist health care practitioners in predicting subjects who would be at a greater risk of developing cardiovascular diseases.

## Background

A particular type of coping or adapting to everyday challenges has been a well-known phenomenon. Coping styles are defined as the patterns of behaviour, thought and emotion mostly used by a person when confronted with new or unusual situations [1]. These conditions could be both good (birth, marriage, getting a promotion, etc.) and negative (death of a loved one, divorce, loss of a job etc.). Cardiovascular disease (CVD) and its risk factors such as hypertension, hyperglycemia, smoking, hyperlipidemia and obesity have been extensively studied. Numerous studies on coping styles with these CVD risk factors have been documented in the literature [2–9]. However, scarcity of data pertaining to coping styles with a biochemical marker, associated with the aforesaid risk factors is striking [10]. Such assessment would certainly assist in predicting individuals who are at a higher risk of developing CVD. Laboratory study of acute stress among 83 undergraduate male students with repressive coping subjects recorded lower high-density lipoprotein (HDL) level and a higher total/HDL cholesterol ratio [2].

Burns et al. [11] used a biochemical marker to demonstrate that acceptance coping was a significant predictor for hepatitis B antibody concentration. Besides, there has been a study exploring the sociodemographic facets of hyperlipidemia [12] which is devoid of relationship with coping styles whatsoever.

By means of HbA1c (glycated hemoglobin) estimation, Graue et al. [3] concluded that adolescents with type 1 diabetes who predominantly employed emotion-focused coping styles had poor metabolic control as well as lower degree of diabetes-related quality of life compared to those utilizing primarily active coping styles. The study by von Kanel et al. [13] among the elderly utilising ways of coping checklist revealed that, predominant engagement of seeking social support was linked to high levels of biochemical markers such as serum amyloid A (SAA), C-reactive protein (CRP), soluble vascular cellular adhesion molecule (sVCAM) -1 and D-dimer thereby increasing the risk of artherothrombotic CVD.

Costantini et al. [14] stated using rodents and exposed them to natural environment to test the interaction between plasma oxidative status, cortisol and coping styles. They identified cortisol as a potential modulator for the different coping styles during baseline and stress-induced plasma oxidative conditions.

Thus, this study was designed to determine the relationship between coping styles and lipid profile among adult staff in a public university using the more established and superior [15] Coping Inventory for Stressful Situations (CISS) questionnaire.

## Methods

### Participants

This was a cross-sectional study enrolling all staff (both academic and non-academic) who were stratified according to race and gender from a tertiary education centre based on the staff registry list. Subsequently, each subject was contacted to ascertain their medical history. Inclusion criteria were all subjects aged 18 years or older and fluent in both the Malay or English languages. Exclusion criteria included those having any form of medical diseases, body mass index (BMI) of more than 30 kg/m^2^ and a family history of hypercholesterolemia.

**Fig. 1:**
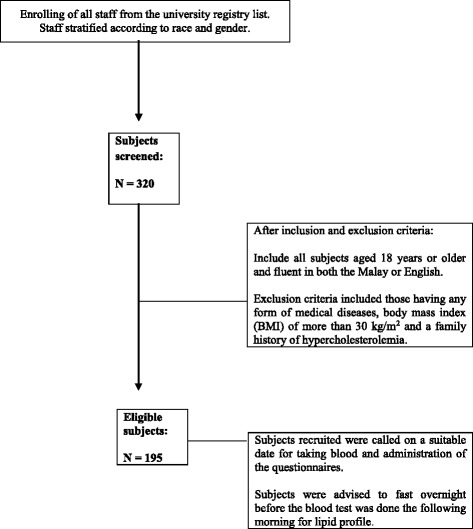
Study flow chart

The eligible subjects were called on a suitable date for taking blood and administration of the questionnaires. These subjects were advised to fast overnight before the blood test was done the following morning for total cholesterol (TC), triglyceride (TG), HDL-cholesterol (high density lipoprotein-cholesterol) and LDL-cholesterol (Low density lipoprotein-cholesterol), collectively known as lipid profile (Fig. 1). Informed consent was obtained from the subjects prior to the blood taking.

Subjects’ details were obtained pertaining to sociodemography (such as staff category, gender, race, marital status and highest education level attained), coping styles and lipid profile.

This project was approved by the University Teknologi MARA, Research and Ethics Committee, reference number 100-FF (19/1/1).

### Questionnaire and definitions

Coping styles were assessed using the validated [16] Coping Inventory for Stressful Situations (CISS) questionnaire which evaluated 3 types of coping styles namely task, emotion and avoidance. It is a self-reported instrument developed by Endler and Parker [17, 18] measuring the subjects coping styles when faced with stressful situations. It is a 48 item tool of a five point Likert-type rating scale ranging from (1) “Not at all” to (5) “Very much.” Elevated levels of TC, TG, LDL cholesterol and reduced level of HDL-cholesterol were classified as abnormal based on NCEP-ATP III [National Cholesterol Education Program (NCEP) Expert Panel on Detection, Evaluation, and Treatment of High Blood Cholesterol in Adults (Adult Treatment Panel III)], 2002 definition [19].

### Statistical analyses

Data was analysed using the Statistical Package for Social Sciences (SPSS) version 16 computer program [20]. For the sociodemographic characteristics, descriptive analysis such as frequency, mean, median and range was used. Chi square was used for discrete variable and Student T-test for continuous variables to determine the various relationships. Pearson’s correlation coefficient (r) was used to measure the strength of association between lipid profile and coping styles. A *p*-value < 0.05 was considered to be statistically significant.

## Results

A total of 320 subjects were screened during the study period and 195 were eligible (61%). The characteristics of the subjects who are eligible are depicted in Table 1. The results showed that majority of subjects were non-academic staff (60.0%), female (67.2%), Malay (91.8%), married (52.3%) and those who had a diploma level of education (34.9%).

**Table 1:** Demographic profile of the study subjects

Demographic profile (*n* = 195)		Number [%] of subjects
Staff category	Academic	78 (40.0)
	Non Academic	117 (60.0)
Gender	Male	64 (32.8)
	Female	131 (67.2)
Race	Non Malay	16 (8.2)
	Malay	179 (91.8)
Marital status	Married	102 (52.3)
	Single	89 (45.6)
	Divorced	2 (1.0)
	Widow/ Widower	2 (1.0)
Highest education attained	Secondary	32 (16.4)
	Diploma	68 (34.9)
	Bachelor	25 (12.8)
	Master	52 (26.7)
	PhD	18 (9.2)

It was observed from Table 2 that there was no significant difference in the percentage of abnormal lipid profile between academic and non academic staff. Apart from TC, males were found to have a significantly higher percentage of abnormal HDL, LDL and TG levels compared to females. Furthermore, pertaining to race it was observed that there were no significant differences with regards to TC, HDL and LDL, whilst abnormal TG was significantly lower among Malays compared to non-Malays. In terms of education, there was no significant difference in the lipid profile among the different categories of educational level. For marital status, it was revealed that married individual had a significantly higher percentage of abnormal TC, HDL, LDL and TG compared to single/divorced/widowed subjects.

**Table 2:** Lipid profile distribution by socio-demography characteristics

Demographic characteristics (*n* = 195)		Total Cholesterol		High Density Lipoprotein		Low Density Lipoprotein		Triglycerides	
		Number [%] of subjects							
		Normal	Abnormal	Normal	Abnormal	Normal	Abnormal	Normal	Abnormal
Staff	Academic	37(47.4)	41(52.6)	68(87.2)	10(12.8)	50(64.1)	28(35.9)	62(79.5)	16(20.5)
	Non-academic	55(47.0)	62(53.0)	110(94.0)	7(6.0)	73(62.4)	44(37.6)	98(83.8)	19(16.2)
	*p* value	0.953		0.097		0.809		0.446	
Gender	Male	27(42.2)	37(57.8)	51(79.7)	13(20.3)	34(53.1)	30(46.9)	41(64.1)	23(35.9)
	Female	65(49.6)	66(50.4)	127(96.9)	4(3.1)	89(67.9)	42(32.1)	119(90.8)	12(9.2)
	*p* value	0.329		0.000^*^		0.044^*^		0.000^*^	
Race	Non -Malay	8(50.0)	8(50.0)	15(93.8)	1(6.2)	11(68.8)	5(31.2)	10(62.5)	6(37.5)
	Malay	84(46.9)	95(53.1)	163(91.1)	16(8.9)	112(62.6)	67(37.4)	150(83.8)	29(16.2)
	*p* value	0.814		0.715		0.624		0.033^*^	
Education	Secondary	17(53.1)	15(46.9)	28(87.5)	4(12.5)	19(59.4)	13(40.6)	23(71.9)	9(28.1)
	Diploma	32(47.1)	36(52.9)	66(97.1)	2(2.9)	43(63.2)	25(36.8)	61(89.7)	7(10.3)
	Bachelor	12(48.0)	13(52.0)	22(88.0)	3(12.0)	14(56.0)	11(44.0)	21(84.0)	4(16.0)
	Master/PhD	31(44.3)	39(55.7)	62(88.6)	8(11.4)	47(67.1)	23(32.9)	55(78.6)	15(21.4)
	*p* value	0.874		0.220		0.747		0.133	
Marital status	Married	37(36.3)	65(63.7)	89(87.3)	13(12.7)	54(52.9)	48(47.1)	76(74.5)	26(25.5)
	Single/divorced/widowed	55(59.1)	38(40.9)	89(95.7)	4(4.3)	69(74.2)	24(25.8)	84(90.3)	9(9.7)
	*p* value	0.001^*^		0.037^*^		0.002^*^		0.004^*^	

*p value is significant at 0.05

Table 3 presents the mean and standard deviation of coping categories score by staff, race and marital status based on CISS. Academic staff scored significantly higher mean scores in task oriented coping styles compared to non-academic staff. In contrast, non-academic staff scored significantly higher mean scores in emotion and avoidance-oriented coping styles compared to academic staff. Malay subjects were found to have significantly higher mean scores in emotion and avoidance-oriented coping styles compared to non-Malay subjects. On the other hand, non-Malay subjects were found to have significantly higher mean scores in task-oriented coping styles compared to Malay subjects. Single/divorced/widowed individuals were found to have significantly higher mean scores in emotion and avoidance-oriented coping styles compared to married individuals. Pertaining to task-oriented coping styles there were no significant differences between the two categories.

**Table 3:** Mean and standard deviation of coping categories score by staff, ethnicity and marital status

		Coping categories					
Demographic characteristics		Task		Emotional		Avoidance	
		Mean	SD	Mean	SD	Mean	SD
Staff	Academic	64.12	7.54	43.86	9.63	49.53	10.73
	Non-academic	61.49	6.39	48.05	8.79	57.61	8.84
	*p* value	0.010*		0.002*		0.000*	
Race	Non -Malay	66.00	6.24	37.81	8.34	44.81	10.00
	Malay	62.23	6.97	47.14	9.058	55.23	10.03
	*p* value	0.038*		0.000*		0.000*	
Marital status	Married	62.42	7.38	44.77	9.03	52.11	11.37
	Single/divorced/widowed	62.67	6.54	48.13	9.40	56.86	8.63
	*p* value	0.807		0.012*		0.001*	

*Abbreviation: SD* standard deviation

*p value is significant at 0.05

In the analysis of the linear relationship between the different lipid markers and coping styles (refer Table 4), it was shown that there was a significant negative correlation between TC (*r* = −0.162) and LDL (*r* = −0.168) with avoidance-oriented coping styles (*p* = 0.023, *p* = 0.019 respectively). There was no significant correlation observed between other coping styles with lipid profile parameters.

**Table 4:** Correlation analysis of task, emotion and avoidance-oriented coping styles with lipid profile

Lipid profile Indices		Task-oriented coping	Emotion-oriented coping	Avoidance-oriented coping
Total Cholesterol (mmol/L)	Pearson Correlation	−0.014	−0.114	−0.162^a^
	Sig. (2-tailed)	0.844	0.113	0.023
HDL (mmol/L)	Pearson Correlation	0.007	−0.064	−0.041
	Sig. (2-tailed)	0.925	0.372	0.571
LDL (mmol/L)	Pearson Correlation	−0.035	−0.079	−0.168^a^
	Sig. (2-tailed)	0.628	0.271	0.019
TG (mmol/L)	Pearson Correlation	0.048	−0.074	−0.016
	Sig. (2-tailed)	0.506	0.305	0.826

^a^Correlation is significant at the 0.05 level (2-tailed)

Table 5 shows the factors associated with abnormal lipid profile. It was noted that the older the age, the more likelihood of abnormal TC and LDL obtained. Being male was associated with greater likelihood of abnormal TG and HDL. In contrast, holding a degree higher than secondary level education was associated with less likelihood of abnormal LDL.

**Table 5:** Results of multivariate regression analysis including variables significantly associated with abnormal lipid profile

			B	S.E.	p	OR	95% CI	
							Lower	Upper
TC	Age		0.051	0.022	0.018*	1.053	1.009	1.098
TG	Gender	Female				1		
		Male	1.734	0.437	0.000*	5.662	2.406	13.325
HDL	Gender	Female				1		
		Male	2.326	0.636	0.000*	10.240	2.944	35.614
LDL	Age		0.070	0.022	0.002*	1.072	1.026	1.120
	Education	Secondary				1		
		Above secondary	−0.845	0.426	0.047*	0.430	0.186	0.990

*Abbreviation: OR* odds ratio

*p value is significant at 0.05

## Discussion

To the best of our knowledge, this was the first study assessing the relationship between lipid profile and coping styles among apparently healthy adult subjects in a tertiary education centre. In the light of the literature being inundated with conflicting reports, we believe that the results of this study would shed light to offer better approaches to coping styles.

The result of female gender being more represented is also reflected in the pattern of gender distribution of our university, comprising 60% female and 40% male staff.

Among the races involved, Malays constituted the majority and this finding is in keeping with the overall distribution of races in the university.

This study showed that males have significantly higher percentage of abnormal HDL, LDL and TG compared to females. These results are consistent with that recorded by other authors both locally [12] and abroad [21].

A notable feature in our study affirmed that abnormal TG was significantly lower among Malays compared to non-Malays. This was possibly attributable mainly due to the dietary habits of non-Malays especially the Indians who formed the majority of individuals in this category. Indians generally tend to consume food rich in saturated fat such as coconut milk, ghee, palm kernel and palm oil which consequently, increased the levels of TG [22].

Pertaining to marital status, this study revealed that single/divorced/widowed individuals demonstrated favourable lipid levels and hence, less CVD risks factor profile. This finding is consistent across several countries such as Hong Kong [23] and Iran [24]. The plausible reason for this could be linked to lifestyle patterns among the married subjects who had regrettably assumed a sedentary lifestyle after matrimony. Therefore, this had affected the lipid indices.

In terms of coping styles, academic staff scored significantly higher mean scores in task oriented coping compared to non-academic staff. This finding concurred with a study by Kariv and Heiman [25] which recorded that task oriented coping styles were more likely to be adopted as it was agreeable to their nature of work as an academician. Having better control of their problems, academic staff fared better in terms of their emotion and physical states [26, 27]. In contrast, non academic staff scored significantly higher mean scores in emotion and avoidance-orientated coping styles compared to academic staff. We presume that non academic staff chooses emotion-focused approach because it is a way of helping them to endure the stress effectively.

As regards race, Malay subjects were found to have significantly higher mean scores in emotion and avoidance-orientated coping styles compared to non-Malay subjects. We hypothesized that Malay subjects tended to cope more by using these styles which had assisted them in modulating their stress response to a manageable level. Non-malay subjects were found to have significantly higher mean scores in task orientated coping styles compared to Malay subjects. This could be ascribed to the fact that the non-Malay subjects focused on solving their problem rather than engaging in avoiding or emotional ways of coping styles. Further studies (both qualitative and quantitative) are required to elucidate why Malays tend to choose emotion and avoidance coping styles unlike the non-Malay subjects who utilise task orientated coping styles.

In addition, single/divorced/widowed individuals were found to have significantly higher mean scores in emotion and avoidance-orientated coping styles compared to married individuals. We postulate that these individuals preferred to use emotion oriented coping approach as it can assist them in regulating their emotion while facing stressful situations. A study by Boals [28] suggested that avoidance coping styles were associated with having less self control. Further studies would help understand if this phenomenon is beneficial to them.

As regards the relationship between lipid profile and different coping categories, this study found that there was a significant negative correlation [29] between TC (*r* = −0.162) and LDL (*r* = −0.168) with avoidance-oriented coping styles (*p* = 0.023, *p* = 0.019 respectively). Hence, avoidance-oriented coping style engendered favourable lipid profile with regards to reduction in TC and LDL, thereby a plausible reduction in cardiovascular event or disease.

An intriguing finding from this study identified that significant correlation faded away in the multiple logistic regressions denoting the presence of confounding effect. Admittedly, age was associated with abnormal TC and LDL. Similarly, being a male was an independent risk factor for abnormal TG and HDL. It was expected that higher education level would be associated with better health status. In this study, higher educational level was associated with favourable LDL.

The strength of this study lies on its study design which differed significantly from earlier screening studies [3, 12]. Subjects recruited were not only stratified by race but also included asymptomatic and healthy ones.

Admittedly, few weaknesses merit mention. Firstly, the CISS questionnaire by itself appears modifiable as it did not encompass facets of other ways of coping approaches. For instance, it failed to explore religious ways of coping which were indeed adopted by many individuals residing in a multi religious country like ours. Secondly, our study was confined to a circumscribed population of adult university staff and therefore it cannot be generalized. Thirdly, this being a cross-sectional study, it does not allow for cause and effect relationships to be studied. Fourthly, although the questionnaire was assumed to be valid and had been used in the Malaysian context, the use of questionnaires in general is inherently affected by several issues spanning from difference of understanding among respondent to social desirability bias.

## Conclusions

Pertaining to the use of coping styles and lipid profile, assessment of coping styles would certainly assist health care practitioners in predicting individuals who would be at risk of developing cardiovascular diseases. Promoting avoidance coping styles would in fact be beneficial in terms of producing favourable lipid profile especially in the reduction of TC and LDL. Thus, cardiovascular diseases in the future could possibly be averted. Consequently, this would be potentially life-saving in the long run.

Moreover, the role of nutraceuticals and functional food ingredients in the management of dyslipidaemia should be considered though their actual CVD risk protection is still under much discussion [30].

## Abbreviations

BMI: Body mass index
CISS: Coping Inventory for Stressful Situations
CRP: C-reactive protein
CVD: Cardiovascular disease
HbA1c: Glycated hemoglobin
HDL-C: High-density lipoprotein cholesterol
LDL-C: Low-density lipoprotein cholesterol
NCEP: National Cholesterol Education Program
SAA: serum amyloid A
SPSS: Statistical Package for Social Sciences
sVCAM: Soluble vascular cellular adhesion molecule
TC: Total cholesterol
TG: Triglyceride
